# A rare case of esophageal perforation following a choking episode with complicated course: management and outcomes

**DOI:** 10.1093/jscr/rjaf353

**Published:** 2025-05-30

**Authors:** Alaa R AL-Ihribat, Jamal Ahmad, Yaman N Qunaibi, Anas A Abukhalaf, Anas Alasafrah

**Affiliations:** College of Medicine and Health Sciences, Palestine Polytechnic University, Wadi Al-Hariyah Street, Hebron, Palestine; College of Medicine and Health Sciences, Palestine Polytechnic University, Wadi Al-Hariyah Street, Hebron, Palestine; College of Medicine and Health Sciences, Palestine Polytechnic University, Wadi Al-Hariyah Street, Hebron, Palestine; General Surgery Department, Al-Ahli Hospital, Hebron, 00970, Palestine; Thoracic Surgery Department, Al-Ahli Hospital, Hebron, 00970, Palestine

**Keywords:** esophageal perforation, esophageal stent, stent migration

## Abstract

Esophageal perforation (EP) is a rare yet lethal condition defined as a full-thickness disruption of the esophageal wall, associated with high morbidity and mortality. We describe a 30-year-old man who developed EP following a choking episode while eating meat. Despite initial management with gastroscopy, the patient experienced serious complications such as stents migration and development of broncho-esophageal fistulae. He underwent thoracotomy, debridement, and esophageal repair. He was also treated with esophageal stenting, chest physiotherapy and progressive oral intake. The patient eventually improved and was discharged in stable condition. This case underscores the importance of early diagnosis and proper management of EP, as well as the challenges involved in timely diagnosis and treatment.

## Introduction

Esophageal perforation (EP) is a full-thickness tear of the esophageal wall and represents a surgical emergency with significant risk of morbidity and mortality [1]. Its relatively rare, with an incidence of ~3.1 cases per 1 000 000 population annually [2]. While most cases are iatrogenic, non-iatrogenic causes include malignancy, trauma, caustic ingestion, and foreign body ingestion. Notably, foreign bodies such as bones are common culprits, whereas soft food-induced perforation is uncommon.

Management is complex and varies from surgical to conservative approaches depending on the clinical scenario [3]. We present a rare case of EP due to a soft food bolus, complicated by multiple surgical interventions and stent-related issues, yet ultimately resulting in recovery.

## Case presentation

A 30-year-old male presented to our emergency department after experiencing a choking episode while eating meat 2 hours before arrival. Following that, he tried to drink a small amount of water without significant relief. He subsequently developed abdominal pain, most prominent in the epigastric region and flanks. Due to the family's unreliable history, additional details regarding symptoms and past medical history were limited. The patient had no reported history of shortness of breath, cough, or other systemic complaints.

**Figure 1:**
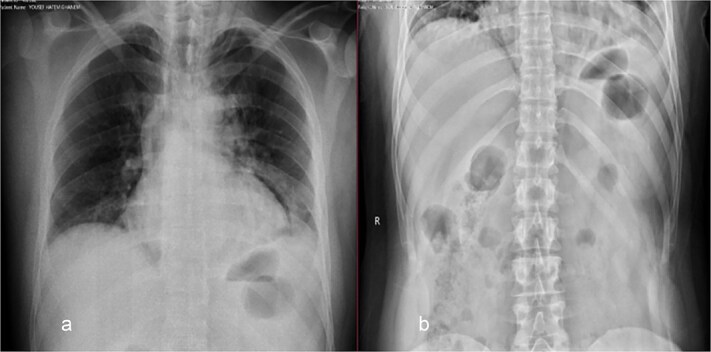
Chest (a) and standing abdomen (b) X-ray: Showed wide mediastinum with pneumomediastinum more at left side and right bronchus bulge. Normal standing abdomen X-ray.

On examination, the patient was vitally stable and not in respiratory distress. His abdomen revealed epigastric tenderness with guarding, with no other significant systemic findings. Abdominal X-ray was normal (Fig. 1). Chest X-ray showed perihilar infiltration and right bronchus bulge (Fig. 1), raising suspicion for aspiration or other pathology. Given the findings, both medical and surgical teams were consulted. A chest and abdominal CT scan with contrast was recommended for further evaluation. However, the family refused and opted for gastroscopy only, despite being informed about the risks.

**Figure 2:**
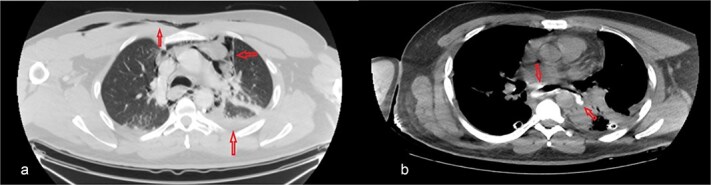
Chest CT scan: (a) with IV contrast, extensive pneumomediastinum as well as extensive cervical and anterior chest subcutaneous surgical emphysema with mild bilateral pleural effusion. (b) IV and oral contrast: Evidence contrast leakage arising from the left posterolateral aspect of mid esophagus associated with mild left loculated pleural effusion and mild pneumomediastinum.

**Figure 3:**
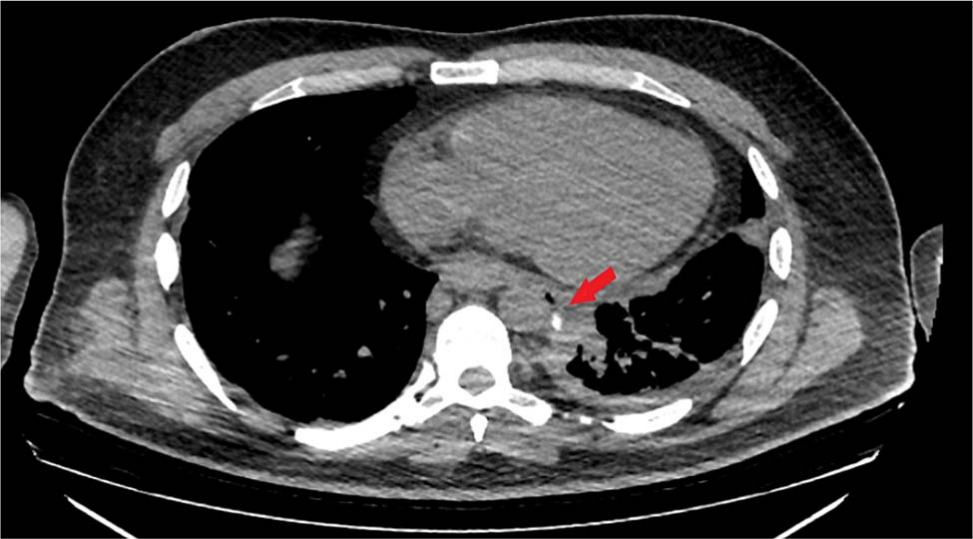
Chest CT scan with oral contrast showing significant reduction in the amount of leaked contrast (small rim remaining) with mild decrease in the amount of left pleural effusion and associated collapse consolidation as well as pneumomediastinum.

The patient was diagnosed with EP. Gastroscopy showed a 2 cm tear at 27–28 cm from the incisors and erosive esophagitis at the gastroesophageal junction (GEJ). Chest CT confirmed pneumomediastinum, subcutaneous emphysema, pleural effusion, and aspiration pneumonia (Fig. 2). The patient was transferred to the SICU for intensive monitoring. On Day 2, a right thoracotomy was performed, revealing a linear esophageal tear, food collection (mainly red meat), and a 1 cm through-and-through perforation. The procedure included debridement, defect repair with pleural patch reinforcement, and chest tube placement. The patient was monitored postoperatively in the SICU. On Day 4, the patient was extubated, and gastroscopy on Day 10 confirmed healing. An esophageal stent was placed on Day 11, with gradual clinical improvement and tolerance of oral fluids. Chest tubes were transitioned to water-seal drainage. Recurrent stent migrations occurred on Days 15, 17, and 19, requiring repositioning and eventual removal. On Day 20, bronchoscopy identified a broncho-esophageal fistula, and chest CT showed reduced leakage (Fig. 3). The patient was kept nothing by mouth (NPO) for further healing.

The patient improved with ambulation, chest physiotherapy, and progressive oral intake. Gastroscopy on Day 28 confirmed healing, and he was discharged on Day 29 in stable condition, tolerating a soft diet.

## Discussion

EP is a life-threatening condition, defined as a transmural rupture of the esophagus [4]. It can result from both iatrogenic and non-iatrogenic causes. Iatrogenic causes constitute 70% of cases, including endoscopy, dilation procedures, and nasogastric tube placement. Non-iatrogenic causes include Boerhaave syndrome, trauma, malignancies, and foreign body ingestion, usually bones: chicken, fish, pigeon, rabbit, and pork [5, 6]. Common sites of injury include the cricopharyngeal sphincter, aortic arch, and GEJ [6]. In our case, the patient presented with a choking episode while eating meat 2 hours before arrival, making it unique, as it did not involve a sharp object typically responsible for thoracic esophageal tears.

Clinical presentation depends on perforation location and time to diagnosis. A review by Chirica *et al.* indicates the following: cervical perforation (27%), which usually presents with subcutaneous emphysema and cervical pain, sometimes with dysphagia, dysphonia, or fever; thoracic perforations (54%) may present with vomiting, thoracic pain, dyspnea, epigastric pain, and dysphagia; abdominal perforations (19%) may present with dorsal or epigastric pain, guarding, and septic shock [7, 8]. Our patient presented with epigastric and flank pain, with no other specific symptoms. This broadened the differential diagnosis, including perforated ulcer, acute pancreatitis, intestinal obstruction or ischemia, EP, and gastric volvulus. Poor reliability of the patient's history also complicated diagnosis.

Diagnosing EP is challenging due to its variable clinical and radiologic presentations. Conventional radiology, such as chest X-ray, is abnormal in 90% of thoracic EPs, showing pleural effusion, pneumothorax, or pneumomediastinum [9]. However, findings may appear only after 1 hour, leading to false negatives if performed too early [7]. In our case, the chest X-ray suggested aspiration rather than EP. Although contrast-enhanced CT is the gold standard due to its high sensitivity (92%–100%) [7], the patient’s family initially refused it in favor of gastroscopy. Gastroscopy confirmed a mid-thoracic esophageal tear, and later chest CT revealed other signs of EP. This highlights the diagnostic complexity of EP, particularly when imaging is atypical or delayed.

Management of EP depends on perforation size, location, patient stability, and timing. Conservative treatment is reserved for stable patients with contained perforations. Early diagnosis in stable patients often involves endoscopic clipping or stenting. Surgical management is required for large, uncontained perforations or unstable patients and may include primary repair, drainage, or esophageal resection. Minimally invasive techniques such as video-assisted thoracoscopic surgery (VATS) are emerging alternatives [6]. In cases with significant fluid leakage, tissue necrosis, or devitalization, as in our case, urgent surgical action is required, including stenting, debridement, primary repair, or drainage [10]. In our case, urgent surgical intervention was needed due to extensive leakage and contamination.

Stents are increasingly used for thoracic EP due to their effectiveness in preventing leaks and reducing mortality and morbidity. However, they carry a risk of migration (7%–75%) [6]. Our patient experienced stent migration three times, requiring repositioning and eventual removal. Securing the stent with sutures has shown success in other reports. Complications can include bleeding, perforation, obstruction, and fistula formation [11]. On Day 20, bronchoscopy identified a broncho-esophageal fistula, so the patient was kept NPO and improved with conservative therapy. Stent removal within 28 days is recommended to reduce complications [11]. Final gastroscopy on Day 28 confirmed healing, and the patient was discharged on Day 29 in stable condition.

In contrast to previously reported cases of EP due to food impaction—most commonly involving sharp objects like fish bones, as described by Munusamy *et al.* [12], Schneider *et al*. [13], and Kimura *et al.* [14]—our case is notable for involving a much younger patient (30 years old) and a soft food item, namely red meat. While surgical repair and esophageal stenting remains the cornerstone of EP management, alternative techniques such as fibrin glue have been explored in select cases, including the chronic thoracic-esophageal fistula reported by Kimura *et al*. [10] In our patient, despite appropriate stent placement, repeated migration occurred without a clear mechanical or anatomical cause. This highlights the need for further investigation into stent stability and long-term outcomes, particularly in younger individuals with non-sharp food-related EP.

## Conclusion

EP is a complex condition that should be diagnosed and treated quickly. While conservative management with endoscopic stenting is sufficient for stable cases, surgical repair is indicated for perforations that are large or not contained. This case highlights the challenges associated with the diagnosis of EP, especially in the presence of atypical radiological features. It also emphasizes the challenges posed by stenting (migration and broncho-esophageal fistula formation), which require a lot of care. Early recognition, timely intervention, and appropriate follow-up care are critical to achieving favorable outcomes in patients with EP.

## Data Availability

The data that support the findings of this study are available from corresponding author upon reasonable request.
